# subMG automates data submission for metagenomics studies

**DOI:** 10.1186/s13040-025-00453-w

**Published:** 2025-06-05

**Authors:** Tom Tubbesing, Andreas Schlüter, Alexander Sczyrba

**Affiliations:** 1 Computational Metagenomics Group, Center for Biotechnology (CeBiTec), Bielefeld University, Universitätsstraße 27, 33615 Bielefeld, Germany; 2 IBG-5: Computational Metagenomics, Institute of Bio- and Geosciences (IBG), Forschungszentrum Jülich GmbH, c/o Centrum für Biotechnologie (CeBiTec), 33594 Bielefeld, Germany

**Keywords:** Metagenomics, European Nucleotide Archive, Submission, FAIR, Metadata

## Abstract

**Background:**

Publicly available metagenomics datasets are crucial for ensuring the reproducibility of scientific findings and supporting contemporary large-scale studies. However, submitting a comprehensive metagenomics dataset is both cumbersome and time-consuming. It requires including sample information, sequencing reads, assemblies, binned contigs, metagenome-assembled genomes (MAGs), and appropriate metadata. As a result, metagenomics studies are often published with incomplete datasets or, in some cases, without any data at all. subMG addresses this challenge by simplifying and automating the data submission process, thereby encouraging broader and more consistent data sharing.

**Results:**

subMG streamlines the process of submitting metagenomics study results to the European Nucleotide Archive (ENA) by allowing researchers to input files and metadata from their studies in a single form and automating downstream tasks that otherwise require extensive manual effort and expertise. The tool comes with comprehensive documentation as well as example data tailored for different use cases and can be operated via the command-line or a graphical user interface (GUI), making it easily deployable to a wide range of potential users.

**Conclusions:**

By simplifying the submission of genome-resolved metagenomics study datasets, subMG significantly reduces the time, effort, and expertise required from researchers, thus paving the way for more numerous and comprehensive data submissions in the future. An increased availability of well-documented and FAIR data can benefit future research, particularly in meta-analyses and comparative studies.

## Background

In modern genome-resolved metagenomics studies, large amounts of data are generated in the form of sequencing reads, assemblies, binned contigs, metagenome-assembled genomes (MAGs), and annotations. The European Nucleotide Archive (ENA) encourages submission of all these data [1] (enriched with appropriate metadata), which is also mandatory when following the FAIR principles (Findable, Accessible, Interoperable, Reusable) [2]. Unfortunately, one fifth of metagenomics studies do not provide any sequencing data at all [3]. Additionally, we often observe that even when raw sequencing reads are deposited in public repositories, complementary processed data – such as assemblies, bins, or MAGs – are not consistently shared.

Presumably, one of the reasons for this situation is that data upload requires expertise and a substantial time investment, often at a point in time when work on the study is mostly finished and researchers are eager to wrap up the publication process. When considering single-organism studies, the complexity of submission is limited, and the work can be carried out manually using the ENA’s submission web interface for most steps. With metagenomic studies, however, the task becomes more complex. Metadata have to be entered for numerous different objects, with a lot of information having to be provided multiple times at different points in the submission process. There are different avenues for submitting this metadata, namely uploading spreadsheets, uploading XML files or submitting tab-separated manifest files through the Webin-CLI tool [4]. No single option works for all the data relevant to a metagenomics study. Accordingly, the user is forced to use at least two different methods. Apart from the submission of samples, reads, and an assembly, it is generally desirable to submit collections of binned contigs (bins) as well. Researchers might furthermore want to submit high-quality bins as MAGs. The *Minimum Information about a Metagenome-Assembled Genome* (MIMAG) standard [5] offers guidelines on how to differentiate the most high-quality bins for this purpose. MAG submission involves first creating virtual sample objects for each bin/MAG and subsequently uploading genomic information. When uploading (meta)data, users have to keep track of any accessions assigned by ENA since these need to be referenced in downstream submission steps (e.g. virtual bin sample accessions have to be referenced when uploading the corresponding MAGs).

In addition, it is challenging for researchers to determine which metadata are required at the outset of the submission process. For example, some information is mandatory for MAG submission but optional when submitting samples and assemblies. Since samples must be submitted first, a user might initially omit metadata that later needs to be gathered and submitted anyway.

Determining the correct taxonomic identifiers for each metagenomic bin is another potentially time-consuming task. This is because taxonomic classification software typically does not output the environmental organism-level taxonomy required for submission. For example, a genome classified at family level as *Enterobacteriaceae* (taxonomic identifier 543) and without a genus-level classification needs to be submitted as uncultured Enterobacteriaceae bacterium (taxonomic identifier 218034).

As microbiome studies routinely yield hundreds of bins, manual submission becomes unfeasible. Furthermore, manual steps increase the likelihood of inadvertently submitting erroneous data. subMG addresses the aforementioned challenges by automating many of the previously described tasks, requiring user interaction only during the initial submission setup.

## Implementation

subMG is a command-line tool that comes with an optional graphical user interface (GUI) mirroring all functionality of the command-line interface (CLI). The tool is developed in Python, leveraging ENA’s Java-based Webin-CLI [4] for several submission tasks. The inclusion of the latter is dictated by Webin-CLI being the only avenue for submitting genome assemblies to the ENA at the time of writing. subMG is designed to streamline the inherently fragmented and complex process of metagenomics data submission, consolidating various tasks into a single, automated workflow.

A major hurdle in the submission of metagenomics studies is that metadata have to be entered and uploaded in several different places, requiring a lot of redundant data entry. subMG addresses this issue by collecting data in a single document. To use subMG, a user must first specify what kind of data they want to submit using the subMG makecfg command. This will create a form in which the user can then enter necessary metadata as well as the location of all files needed for submission. The form only contains fields that are mandatory for the specific type of submission; however, additional fields can easily be added at the user’s discretion. Inline comments for each field explain what data is required and provide examples. subMG reads information like bin quality, taxonomic assignments, and coverage from common formats such as the outputs generated by CheckM [6] or GTDB-Tk [7] and binary alignment maps (BAMs).

The subMG submit command uses a filled-out configuration file as input, alongside arguments specifying the data levels (samples, reads, assembly, binned contigs, MAGs) to submit. Users can choose between submitting to the production or development services of ENA. Before submission starts, the configuration form and the files referenced therein are validated and potential issues are reported back to the user.

If validation is successful, coverage and taxonomy information are inferred where necessary. subMG queries the ENA taxonomy API to find suitable taxonomies for all bins/MAGs. This assignment can fail, for example in cases where no suitable uncultured taxonomic identifier exists yet. In such cases, subMG halts the submission process and provides the user with a list of problematic bins or MAGs. The user can then address the issue by manually defining the taxonomy for these entries in the configuration form or by requesting novel taxon identifiers using the ENA web portal.

After these preprocessing steps, the submission is carried out. The steps involved in this task depend on the levels of data the user wants to submit. XML files with metadata are created and submitted through an ENA API for steps necessitating the creation of (virtual) sample objects (submission of samples, co-assemblies, binned contigs, MAGs). Receipts returned by the ENA API are parsed to identify issues and extract accession numbers for the downstream submission steps and final reporting. For steps which entail file upload (submission of reads, assemblies, binned contigs, MAGs), a manifest file with item-specific metadata is written and staged alongside the file. Subsequently, it is passed to the Webin-CLI software for upload. Receipts produced by Webin-CLI are parsed similarly to those returned by the aforementioned API.

**Fig. 1:**
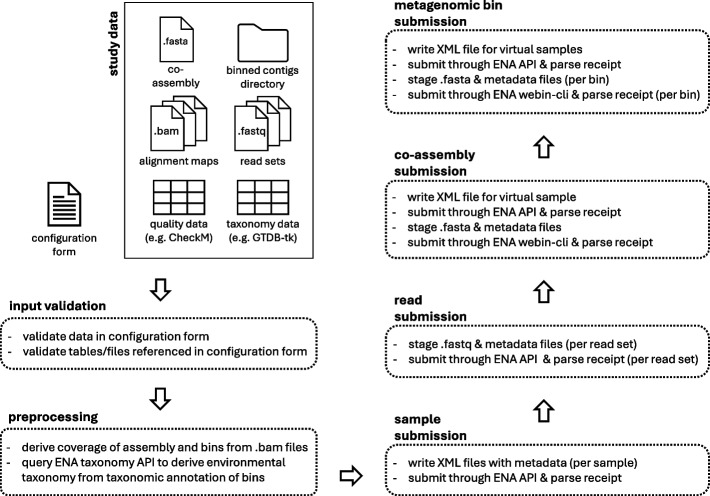
Schematic overview of the submission process carried out by subMG for a scenario where a user wants to submit samples, sequencing reads, a co-assembly and binned contigs

In a typical metagenomics study, a user might want to submit samples, sequencing reads, a co-assembly, and binned contigs. Figure 1 illustrates the steps carried out by the tool in such a case.

Recognizing that not all users are comfortable with command-line tools, subMG also includes a graphical user interface (GUI) which integrates the functionalities of the makecfg and submit commands.

## Results and discussion

subMG significantly simplifies the complex and error-prone process of submitting metagenomic study datasets to the European Nucleotide Archive. The tool enables researchers to complete submissions with reduced time investment while drastically reducing the expertise needed for the task.

**Fig. 2:**
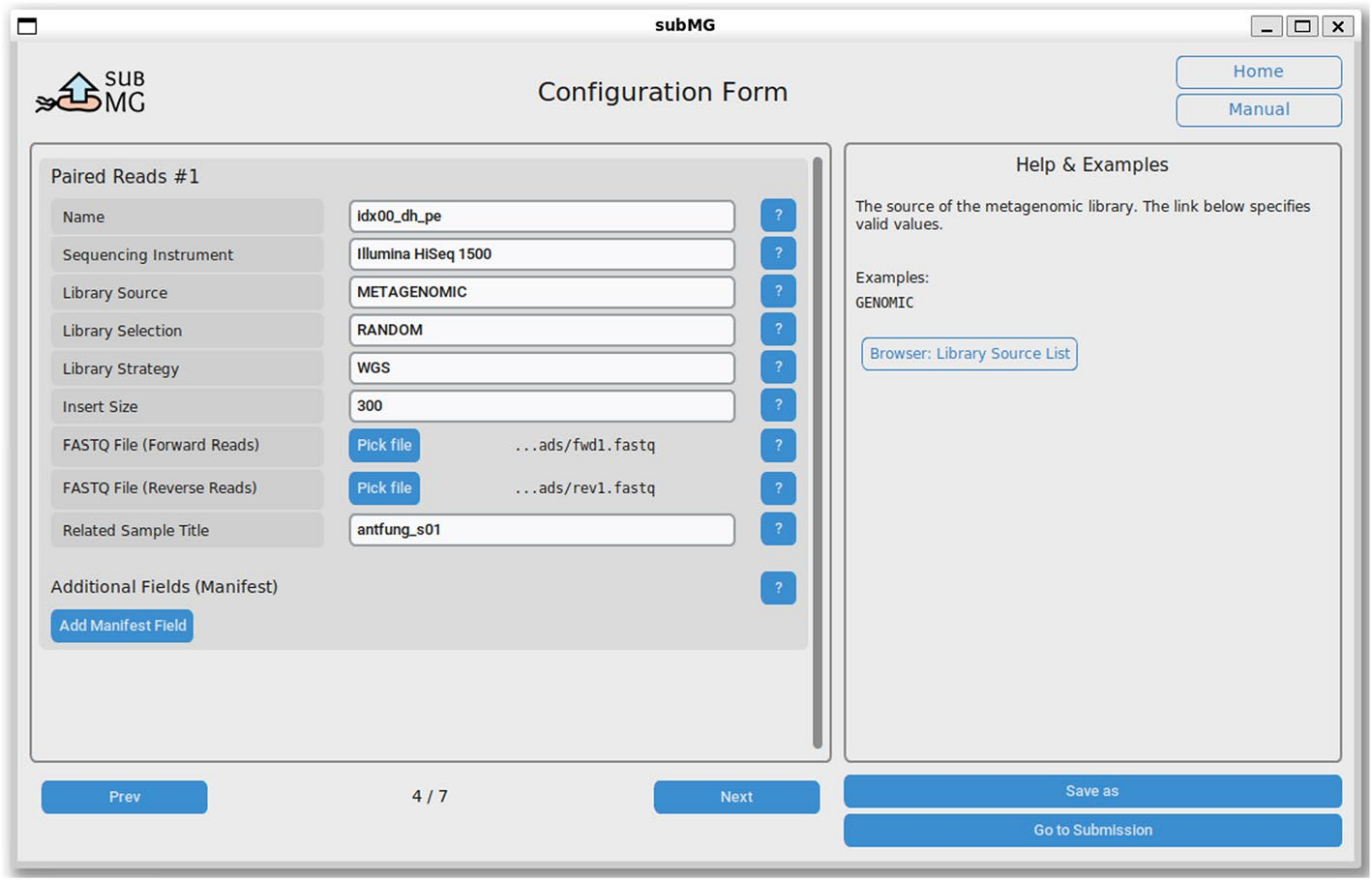
subMG graphical user interface (GUI) for paired-end sequencing reads data entry. The interface allows users to select files and enter mandatory metadata. Help buttons display explanations and examples for each field. Additional metadata can be added at the user’s discretion

To accommodate diverse user preferences, subMG provides a graphical user interface (GUI) in addition to its command-line interface. The GUI allows users to specify the types of data they wish to submit, such as samples, sequencing reads, (co-)assemblies, binned contigs, or MAGs, and then renders custom forms with the required fields (see Fig. 2). These forms include detailed explanations and examples for each field, ensuring that users have the necessary guidance throughout the process.

This functionality mirrors the command-line workflow, which also centers around a comprehensive configuration file. Whether using the GUI or the command line, subMG generates tailored templates that contain only the metadata fields relevant to the user’s chosen submission scenario. Redundant data entry is eliminated, and users can add optional metadata fields at their discretion. While this is intended to lower the barrier of data submission, it would be desirable to encourage users to enrich submissions with additional metadata. Future development of this software will therefore strive to provide study-appropriate suggestions for relevant data fields.

subMG further permits users to extend an existing study when some components (e.g. samples and sequencing reads) have already been deposited. In such cases, the forms are adjusted to request the previously assigned accessions, and the tool will retrieve associated information from the ENA.

To help new users, subMG comes with pre-filled configuration forms and toy datasets, serving as examples for the most common use cases. These forms can be opened via the GUI or inspected in a text editor to help users understand and experiment with different submission scenarios. A tutorial hosted on GitHub Pages teaches users how to operate the CLI tool using the toy datasets. Additionally, they serve as tests for a continuous integration (CI) workflow.

Several other tools assist with ENA submissions, but their scopes differ markedly from that of subMG. For example, EMBL2checklists [8] is built to convert European Molecular Biology Laboratory (EMBL) and GenBank flat files to the ENA *checklist* spreadsheet format for easy submission of barcoded plant and fungal sequences. The tool targets single-isolate or single-gene entries and is not intended to handle the issues of prokaryote metagenome submission outlined above. METAGENOTE [9] provides a web interface to capture sample metadata based on the “Minimum Information about any Sequence” (MIxS) standard [10]. While METAGENOTE greatly simplifies the annotation of metagenomic sample data and ensures standardized metadata, it is limited to the initial stage of submission (samples and sequence reads) and does not support subsequent deposition of assemblies or binned genomes. The Galaxy ENA upload tool [11] similarly streamlines the upload of sequencing reads from within the Galaxy analysis environment, covering study, sample and run objects, but it does not cover additional data layers (assemblies, binned contigs, MAGs). The ISA software suite [12] takes a more general approach. It is focused on metadata management, enabling researchers to describe complex studies with multiple assays. The flexibility of the suite allows it to represent even elaborate metagenomics experiment structures, and users can export metadata in formats compatible with ENA. However, it does not offer an end-to-end process for data upload and carrying out an ENA metagenomics submission still demands considerable configuration and manual conversion. In short, these tools solve important problems, but many of the difficulties specific to metagenomics submission remain unaddressed. By consolidating data entry, integrating every submission layer and automating taxonomy assignment, subMG fills the gap left by existing software and services.

Compared to the default ENA submission workflow, subMG offers a more integrated and user-friendly approach. The standard process often requires redundant metadata entry across multiple forms and necessitates switching between different avenues for data submission/upload. This fragmentation not only increases the likelihood of errors but also demands substantial manual effort. In contrast, subMG consolidates all metadata and file references into a single configuration file, ensuring consistency and reducing the time required for data preparation.

By addressing key barriers in the metagenomics submission workflow and enabling the publication of well-documented datasets, subMG empowers researchers to adhere more closely to FAIR standards, promoting better data accessibility and reuse.

## Conclusions

subMG simplifies the submission of metagenomics datasets by consolidating metadata entry and automating complex tasks, drastically reducing the time and expertise required for ENA submissions. For example, environmental organism-level taxonomies are automatically derived from the taxonomic information commonly produced by annotation pipelines, drastically reducing manual effort. The software handles tracking and referencing accessions of the hundreds of items typically involved in a metagenomics submission, thereby reducing workload and minimizing the potential for human error.

The availability of a GUI as well as a succinct tutorial for the CLI ensures that the tool is accessible to researchers with diverse levels of technical expertise. By making it easier for researchers to create comprehensive and well-documented public datasets, subMG facilitates more consistent data sharing and contributes to the broader goals of FAIR data within the scientific community. This tool represents a significant step towards improving the accessibility and reproducibility of metagenomics research, ensuring that valuable datasets are preserved and made available for future studies.

## Availability and requirements

**Project name:** subMG

**Project home page:** https://github.com/metagenomics/subMG

**Operating system(s):** Platform independent

**Programming language:** Python

**Other requirements:** Python 3.8 or higher, Java 17 or higher

**License:** MIT

## Data Availability

No datasets were generated or analysed during the current study.

## Abbreviations

MAG: Metagenome-Assembled Genome
ENA: European Nucleotide Archive
FAIR: Findable, Accessible, Interoperable, Reusable
XML: Extensible Markup Language
BAM: Binary Alignment Map
API: Application Programming Interface
CLI: Command-Line Interface
EMBL: European Molecular Biology Laboratory
CI: Continuous Integration
